# Targeting the cholesterol-RORα/γ axis inhibits colorectal cancer progression through degrading c-myc

**DOI:** 10.1038/s41388-022-02515-3

**Published:** 2022-10-31

**Authors:** Ying-Nan Wang, Dan-Yun Ruan, Zi-Xian Wang, Kai Yu, Dai-Lin Rong, Ze-Xian Liu, Feng Wang, Jia-Jia Hu, Ying Jin, Qi-Nian Wu, Heng-Ying Pu, Min Wang, Rui-Hua Xu, Zhao-Lei Zeng

**Affiliations:** 1 Department of Medical Oncology, Sun Yat-sen University Cancer Center, State Key Laboratory of Oncology in South China, Collaborative Innovation Center for Cancer Medicine, Sun Yat-sen University, Guangzhou, 510060 China; 2 Research Unit of Precision Diagnosis and Treatment for Gastrointestinal Cancer, Chinese Academy of Medical Sciences, Guangzhou, 510060 China; 3 Department of Clinical Research, State Key Laboratory of Oncology in South China, Collaborative Innovation Center for Cancer Medicine, Sun Yat- Sen University Cancer Center, Guangzhou, China; 4 State Key Laboratory of Oncology in South China, Sun Yat-sen University Cancer Center, Collaborative Innovation Center for Cancer Medicine, Sun Yat-sen University, Guangzhou, 510060 China; 5 Department of Radiology, Third Affiliated Hospital of Sun Yat-Sen University, No. 600, Tianhe Road, Guangzhou, Guangdong 510630 China; 6 Department of Pathology, Sun Yat-sen University Cancer Center; State Key Laboratory of Oncology in South China; Collaborative Innovation Center for Cancer Medicine, Guangzhou, Guangdong, 510060 China

**Keywords:** Colorectal cancer, Cell growth

## Abstract

Dysregulated cholesterol metabolism is a hallmark of colorectal cancer (CRC). However, the usage of cholesterol-lowering agents seemed to have no benefit in CRC patients. In this study, we focused on the cholesterol-nuclear receptors (NRs) axis as a strategy. Cholesterol and its derivatives work as ligands for different nuclear receptors, thus promoting cancer progression. The key NR downstream of cholesterol in CRC is unknown. Here, we treated CRC cells with a cholesterol-lowering agent and lipoprotein-depleted conditioned medium, and then detected the change of the putative NRs. The results revealed that RORα/γ (Retinoic acid receptor-related Orphan Receptor α/γ) levels exhibited the most obvious increases in CRC cells subjected them to cholesterol deprivation. RORα/γ agonists significantly inhibited CRC cells proliferation and migration in vitro and in vivo. Also, RORα/γ overexpression repressed CRC cells proliferation and migration in vitro and in vivo and RORα/γ knockdown promoted it. Mechanistically, RORα/γ agonists promoted c-myc degradation by activating the transcription of the ubiquitinase NEDD4. Intriguingly, the combination of RORα/γ agonists and atorvastatin had a synergistic effect on inhibiting CRC cells. These findings demonstrate that the cholesterol- RORα/γ axis is important for maintaining c-myc protein levels. Combination therapy with atorvastatin and RORα/γ agonist is a promising therapeutic strategy for CRC.

## Abstract (web summary)

Graphical representation of the regulation of c-myc by the cholesterol-RORα/γ axis. Cholesterol in CRC cells is derived from de novo synthesis and exogenous uptake of LDL. Cholesterol and its derivatives inhibit the transcriptional activity of RORα/γ on NEDD4, which results in the stabilization of the c-myc protein. The combination of atorvastatin and SR1078 has a synergistic inhibitory effect on CRC cells.

## Graphical abstract

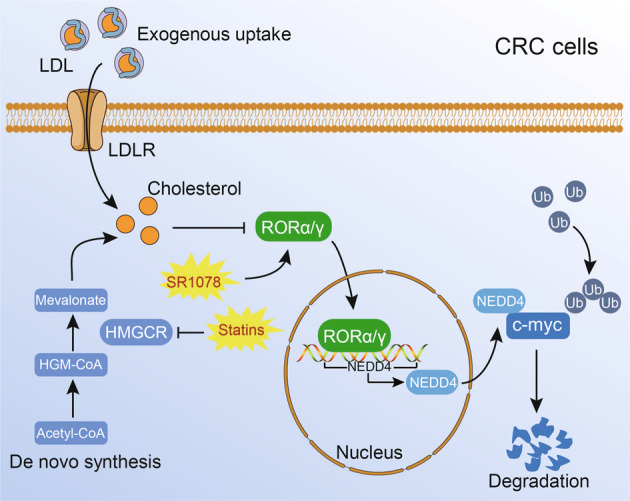

## Introduction

Cholesterol is an essential lipid in the human body. It is obtained by exogenous uptake or endogenous synthesis predominantly in the liver. Cholesterol is water-insoluble and thus is transported throughout the bloodstream in low-density lipoprotein (LDL)-bound form [1]. Cholesterol has many biological functions; it not only is a constituent of the plasma membrane but also constitutes an energy source, participates in signal transduction and regulates inflammation [2, 3]. Dysregulated cholesterol metabolism is a hallmark of cancer development [4, 5]. Indeed, high serum levels of cholesterol are associated with an increased risk of colorectal cancer (CRC) [6]. Cholesterol accumulation is a general feature of cancer tissues, including CRC tissues [7]. Therefore, cholesterol-lowering strategies have therapeutic potential for CRC prevention and treatment.

Statins, which inhibit 3-hydroxy-3-methylglutaryl-coenzyme A (HMG-CoA) reductase, are the most widely prescribed cholesterol-lowering agents for treating cardiovascular and cerebrovascular diseases [8, 9]. Studies have indicated that statins might also have an anti-cancer effect, particularly in CRC. In 2010 and 2020, Bardou M. et al. and Dobrzycka M. et al., respectively, systematically reviewed the research on statins in the chemoprevention, incidence and treatment of CRC [10, 11]. Although accumulating evidence shows that statins have played a positive role in CRC prevention and treatment during those ten-year spans, the stances on the clinical guidelines for the use of statins to treat CRC remain controversial. Notably, the addition of simvastatin to the XELIRI/FOLFIRI regimens did not improve progression-free survival in patients with previously treated metastatic CRC [12]. This finding shows that the combination of statins and chemotherapy does not provide additional benefits in patients and indicates the need for further research to elucidate the mechanism of dysregulated cholesterol metabolism in CRC cells.

Recent research indicates that the products of cholesterol oxygenation, the oxysterols, play an important role in cancer progression [13, 14]. Cholesterol formation is catalyzed by oxidoreductases, hydrolases and transferases under modulation by reactive oxygen species (ROS) and is then converted to oxysterols, including hydroxycholesterol and epoxy cholesterol [15]. Oxysterols can bind to different nuclear receptors (NRs), such as the liver X receptors (LXR), the estrogen receptor (ER) and the retinoic acid receptor-related orphan receptor (ROR), and activate or inactivate them [16, 17]. The function of the cholesterol-NR axis in cancer progression is still unclear. Among the above-mentioned NRs, RORs are members of the orphan nuclear receptor family, a group of NRs that can activate transcription by recruiting coactivators without binding ligands [18]. RORs have three forms: RORα, RORβ and RORγ. RORα and RORγ are widely expressed in many tissues, and RORβ exhibits restricted distribution in the central nervous system, retina and pineal gland. Although RORs were initially identified as “orphan” receptors, some endogenous ligands have been found. Cholesterol, cholesterol-3-O-sulphate and several oxysterols act as inverse agonists of RORs by binding to the ligand-binding domain of the receptor [19, 20]. The biological functions of RORs involve the regulation of circadian rhythms, metabolism, immune function and cancer progression [21]. RORα acts as a tumour suppressor in prostate cancer and breast cancer [22–24]. In CRC, RORα inhibits CRC cell proliferation and migration induced by adipocyte-conditioned medium and suppresses angiopoiesis [25]. In addition, RORγ activates cholesterol biosynthesis in triple-negative breast cancer (TNBC) and stimulates androgen receptor (AR) transcription in castration-resistant prostate cancer (CRPC) [26, 27]. However, little is known about the role of RORγ in CRC. The function of the cholesterol-ROR axis in CRC needs further investigation.

In this study, we identified RORα and RORγ as the most highly elevated NRs in CRC cell lines subjected to cholesterol deprivation. RORα/γ inhibited CRC cell growth and invasion by degrading c-myc. The RORα/γ-selective agonists exhibited a synergistic effect with atorvastatin on inhibiting CRC progression both in vitro and in vivo. Therefore, our findings defined the cholesterol-RORα/γ axis as a previously unsuspected master regulator of the c-myc oncogene and as an attractive therapeutic target in CRC.

## Results

### RORα/γ are the main NRs for cholesterol in CRC

**Fig. 1:**
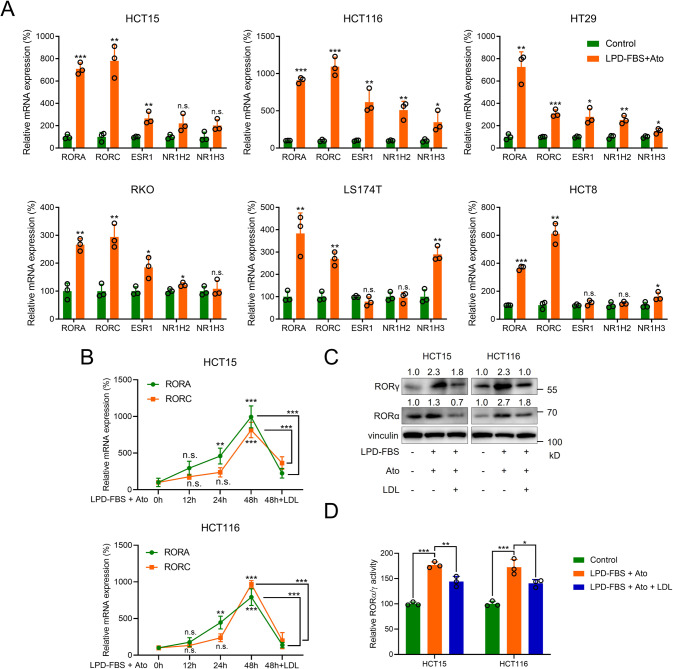
RORα/γ are the main receptors for cholesterol in CRC cells. **A** The relative mRNA expression levels of the RORA, RORC, ESR1, NR1H1 and NR1H3 genes in HCT15, HCT116, HT29, RKO, LS174T and HCT8 CRC cell lines were measured by qRT-PCR (*n* = 3). Cells were subjected to cholesterol deprivation (10 μM atorvastatin and conditioned medium containing LPD-FBS) for 48 h. **B** Relative mRNA expression levels of RORA and RORC in HCT15 and HCT116 cells (*n* = 3). Cells were subjected to cholesterol deprivation or exogenous LDL supplementation at the indicated times. **C**, **D** The protein levels and activities of RORα and RORγ in CRC cell lines were determined by western blot and gene reporter assays (*n* = 3), respectively. Cells were treated with 10 μM atorvastatin and conditioned medium containing LPD-FBS or exogenous LDL supplementation for 48 h. The data are presented as the mean ± SD values. ^*^P < 0.05, ^**^P < 0.01, ^***^P < 0.001; Student’s *t*-test in two groups; One-way ANOVA in more than two groups. Ato atorvastatin, LPD-FBS lipoprotein-depleted foetal bovine serum, LDL low-density lipoprotein.

Because cholesterol and its derivatives are ligands for RORs, ERs and LXRs, we reasoned that NR plays a crucial role downstream of cholesterol in CRC [13, 28]. We thus treated 6 CRC cell lines with atorvastatin and conditioned medium supplemented with LPD-FBS and then measured the mRNA levels of these NRs. Among the NRs, RORA and RORC exhibited greater upregulation than ER (ESR1) and LXRs (NR1H2 and NR1H3) (Fig. 1A). We selected the HCT15 and HCT116 cell lines, which had relatively high fold changes in RORA and RORC expression, for further experiments. In a time-course assay with atorvastatin and conditioned medium supplemented with LPD-FBS, the mRNA levels of RORA and RORC increased gradually from 12 h to 48 h. This upregulation was then inhibited by exogenous LDL supplementation (Fig. 1B). Consistent with this finding, the protein levels of RORα and RORγ exhibited similar changes under cholesterol deprivation and exogenous LDL supplementation (Fig. 1C). As transcription factors (TFs), both RORα and RORγ function by binding the ROR element (RORE) in the promoter region of the target gene. Therefore, we evaluated the transcriptional activity of the RORE by gene reporter assays. Cholesterol deprivation increased RORα/γ activity, and exogenous LDL supplementation decreased RORα/γ activity (Fig. 1D).

### The RORα/γ agonists inhibit CRC growth and metastasis in vitro and in vivo

**Fig. 2:**
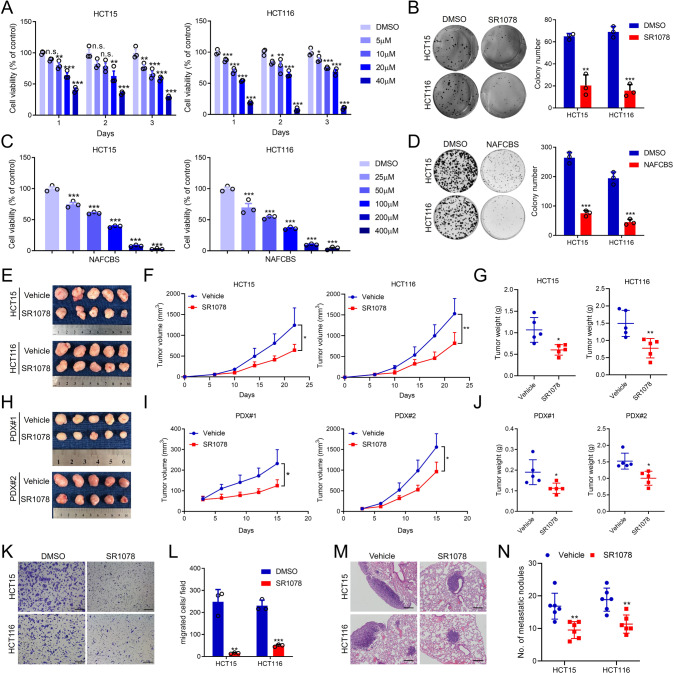
RORα/γ agonists inhibit CRC growth and metastasis in vitro and in vivo. **A** The viability of CRC cell lines was measured with an MTS assay (*n* = 3). Cells were treated with SR1078 at the indicated concentrations and for the indicated times. **B** Colony formation assay of CRC cells treated with DMSO or 10 μM SR1078 (*n* = 3). The colonies were counted. Cell viability (**C**) and colony formation (**D**) was detected in NAFCBS-treated CRC cells. The concentration used in colony formation assay is 100 μM. **E**–**G** Representative images and growth curves of subcutaneous tumours treated with vehicle or SR1078 in nude mice (*n* = 5) (**D**). The tumour weights are shown in **G**. **H**–**J** In two PDX models, the effect of SR1078 on tumour growth was evaluated (*n* = 5). **K**, **L** Transwell assays of CRC cells treated with DMSO or 10 μM SR1078 (*n* = 3). The migrated cells were counted. Scale bars=200 μm. **M**, **N** Nude mice were treated with vehicle or 20 mg/kg SR1078 after tail vein injection of CRC cells (*n* = 6). Lung metastases were evaluated after 8 weeks. Representative H&E staining of lung tissues is shown (**M**), and metastatic nodules were counted (**N**). Scale bars=100 μm. The data are presented as the mean ± SD values. ^*^P < 0.05, ^**^P < 0.01, ^***^P < 0.001; Student’s *t*-test in two groups; One-way ANOVA in more than two groups.

Some molecules targeting RORα and RORγ have been developed [29, 30]. Among these molecules, SR1078 is a RORα/γ-selective dual agonist that activates RORα/γ-driven transcription [31]. And NAFCBS (Compounds 3) is a RORγ agonist that increases SRC1 recruitment [32]. To explore the effect of targeting RORα/γ, we performed a series of experiments to test these agonists. SR1078 inhibited HCT15 and HCT116 cell viability in a concentration-dependent manner. The effect of SR1078 began on the first day and continued until the third day (Fig. 2A). SR1078 also inhibited the colony formation and migration of HCT15 and HCT116 cells at a concentration of 10 μM (Fig. 2B). NAFCBS also inhibited cell viability and colony formation of CRC cells (Fig. 2C, D). Furthermore, SR1078 significantly inhibited tumour growth in nude mice subcutaneously (Fig. 2E–G). In addition, the inhibitory effect of SR1078 on tumour growth was confirmed in two PDX models (Fig. 2H–J). Besides, SR1078 inhibited CRC cells migration in transwell assays and reduced the number of metastatic nodules in lung metastasis models (Fig. 2K–N).

### RORα/γ inhibit CRC growth and metastasis in vitro and in vivo

**Fig. 3:**
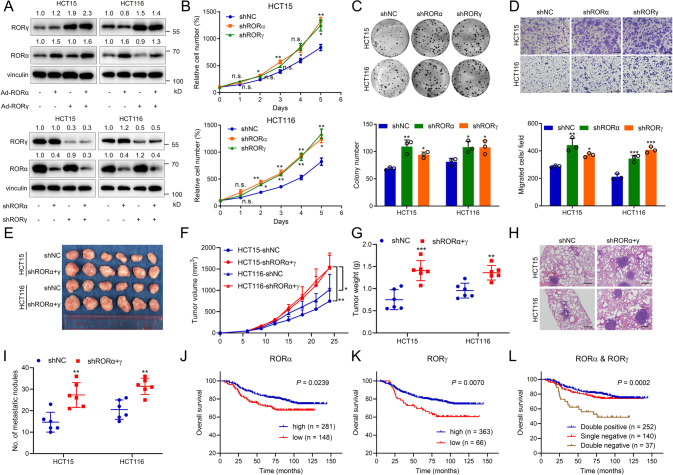
RORα/γ inhibit CRC growth and metastasis in vitro and in vivo. **A** Representative immunoblots showing the RORα and/or RORγ knockdown and overexpression efficiency in CRC cell lines. **B** Growth curves of shNC, shRORα and shRORγ CRC cells, as measured with an MTS assay (*n* = 3). Colony formation assay (**C**) and transwell assay (**D**) of shNC, shRORα and shRORγ CRC cells (*n* = 3). Cells were stained with crystal violet after fixation with methanol. The colonies or migrated cells were counted. Scale bars=200 μm. Representative images (**E**) and growth curves (**F**) of subcutaneous tumours derived from shNC and shRORα + γ cells in nude mice (*n* = 6). Then, the tumours were resected and weighed **G**. **H**, **I** Lung metastasis was evaluated by tail vein injection of shNC and shRORα + γ cells into nude mice. Representative H&E staining of lung tissues is shown (**H**). The metastatic nodules were counted under a microscope (**I**). Scale bars=100 μm. **J**, **K** Kaplan–Meier estimates of survival times for CRC patients with high or low RORα/γ expression in tumour tissues as evaluated by IHC staining. **L** Kaplan–Meier estimates of survival times for CRC patients with different RORα/γ expressions. Double positive: high levels of RORα and RORγ. Single positive: high levels of RORα and low levels of RORγ, or low levels of RORα and high levels of RORγ. Double negative: low levels of RORα and RORγ. The cut-off points for RORα or RORγ were determined by the Youden index using receiver operating characteristic analysis. The data are presented as the mean ± SD values. ^*^P < 0.05, ^**^P < 0.01, ^***^P < 0.001; Student’s *t*-test in two groups; One-way ANOVA in more than two groups. ^*^P < 0.05, ^**^P < 0.01; log-rank test.

To further investigate the function of RORα/γ in CRC cells, we silenced RORα and RORγ by lentiviral transduction and overexpressed them by adenoviral vectors in HCT15 and HCT116 cells and then evaluated the effects of these modulations on malignant cell phenotypes (Fig. 3A). The results of MTS assays and colony formation assays showed that silencing RORα/γ promoted HCT15 and HCT116 cell proliferation and that overexpression of RORα/γ inhibited cell proliferation (Fig. 3B, C and Supplementary Fig. 1A–C). The results of transwell assays showed that the cell invasive ability was enhanced by RORα/γ silencing and decreased by RORα/γ overexpression (Fig. 3D and Supplementary Fig. 1D, E). To confirm the function of RORα/γ in vivo, we subcutaneously injected RORα/γ-overexpressing cells, RORα/γ-silenced cells and the corresponding control cells into nude mice. Tumour growth was significantly accelerated in RORα/γ-silenced cells and slowed in RORα/γ-overexpressing cells (Fig. 3E–G and Supplementary Fig. 1F–H). In addition, we injected control cells and RORα/γ-silenced cells into the tail vein to evaluate their lung metastasis ability. RORα/γ silencing markedly increased the number of metastatic nodules formed from both HCT15 and HCT116 cells (Fig. 3H, I).

To further investigate the influence of RORα/γ on CRC patient survival, we collected tumour tissues from CRC patients and performed immunohistochemical (IHC) analysis of the RORα/γ proteins. Low levels of RORα/γ predicted poor overall survival (OS) of CRC patients (Fig. 2J, K and Supplementary Fig. 2). And patients with low levels of RORα/γ had a poor progression-free survival (PFS) although the *P* value of RORγ is not significant (Supplementary Fig. 3). Particularly, the patients with both low levels of RORα and RORγ had much worse OS and PFS (Fig. 2L and Supplementary Fig. 3).

### The RORα/γ agonist SR1078 promotes c-myc degradation by activating the transcription of the ubiquitinase NEDD4

**Fig. 4:**
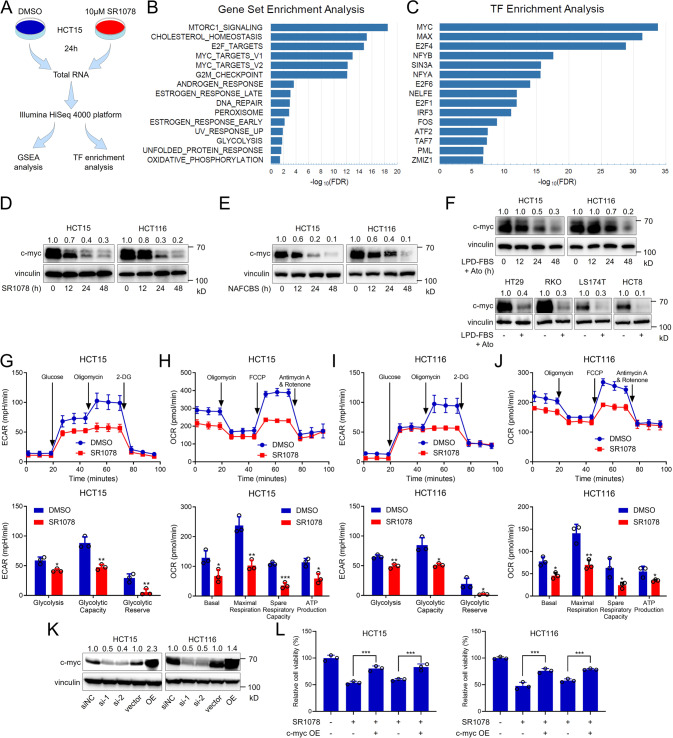
The RORα/γ agonists inhibit c-myc in CRC cells. **A** Cells were treated with DMSO or 10 μM SR1078 for 24 h. Then, total RNA was extracted and sequenced on the Illumina HiSeq 4000 platform. **B**, **C** GSEA and TF enrichment analyses of RNA sequencing data for DMSO-treated and SR1078-treated HCT15 cells (*n* = 3). TF enrichment analysis was performed by Enrichr with the consensus TFs from the ChEA and ENCODE databases. **D**, **E** Representative immunoblotting of c-myc protein levels in HCT15 and HCT116 cells treated with 10 μM SR1078 or 100 μM NAFCBS for 0, 12, 24, or 48 h. **F** Representative immunoblot of c-myc in HCT15 and HCT116 CRC cells treated with atorvastatin and conditioned medium containing LPD-FBS in a time-course assay (upper). Representative immunoblot of c-myc in HT29, RKO, LS174T and HCT8 CRC cells treated with atorvastatin and conditioned medium containing LPD-FBS (lower). **G**–**J** CRC cells were treated with DMSO or 10 μM SR1078 for 24 h. Then, cells were harvested for measurement of the ECAR and OCR using a Seahorse XF24e extracellular flux analyzer (*n* = 3). **K** Immunoblots of c-myc knockdown and overexpression efficiency in HCT15 and HCT116 CRC cell lines. **L** The viability of HCT15 and HCT116 CRC cell lines was evaluated by an ATP assay. MYC-overexpressing CRC cells were treated with DMSO, SR1078 or NAFCBS as indicated for 48 h (*n* = 3). The data are presented as the mean ± SD values. ^*^P < 0.05, ^**^P < 0.01, ^***^P < 0.001; Student’s *t*-test in two groups; One-way ANOVA in more than two groups. TF transcription factor, FDR false discovery rate, OE overexpression.

To understand the mechanism by which SR1078 suppresses malignant cell phenotypes, we performed RNA sequencing on DMSO-treated HCT15 cells and SR1078-treated HCT15 cells (Fig. 4A). The differential analysis of gene expression was shown in Supplementary Table 6. Figure 4B shows the top enriched cancer hallmarks pathways in the DMSO-treated group as determined by gene set enrichment analysis (GSEA). We focused on the MYC targets in the V1 and V2 pathways. Intriguingly, these two gene sets shared only a few genes, but both sets were enriched in DMSO-treated cells. The oncogene MYC is a TF that drives cancer development and progression. Therefore, we performed TF enrichment analysis to explore the TFs influenced by SR1078. MYC was the most strongly affected TF in HCT15 cells treated with SR1078 (Fig. 4C). These results reminded us that RORα/γ agonists might inhibit the malignant phenotype of CRC cells by regulating c-myc. To confirm our hypothesis, we measured the protein levels of c-myc in HCT15 and HCT116 cells treated with SR1078 or NAFCBS in a time-course assay. As shown in Fig. 4D, E, the c-myc protein levels were markedly reduced after SR1078 or NAFCBS treatment. Considering that RORα/γ are inactivated by cholesterol and its derivatives, we then measured c-myc levels upon cholesterol deprivation. The levels of c-myc were reduced time-dependently in HCT15 and HCT116 cells treated with a conditioned medium containing LPD-FBS and atorvastatin (Fig. 4F). Also, the decrease in c-myc levels by cholesterol deprivation was confirmed in four other CRC cell lines, including HT29, RKO, LS174T and HCT8 (Fig. 4F). C-myc levels are critical for processes related to energy metabolism in cancer cells, including glycolysis and mitochondrial respiration. To confirm this, we determined the Seahorse assay using a Seahorse XF24e extracellular flux analyser. We found that both the extracellular acidification rate (ECAR) and oxygen consumption rate (OCR) in c-myc-silenced CRC cells were inhibited (Fig. 4K and Supplementary Fig. 4). Then, we detected the ECAR and OCR in SR1078-treated CRC cells. The results showed that both glycolysis and mitochondrial respiration were significantly inhibited in CRC cells by SR1078 (Fig. 4G–J). What’s more, c-myc overexpression could rescue cell viability decreased by SR1078 or NAFCBS treatment (Fig. 4K, L).

**Fig. 5:**
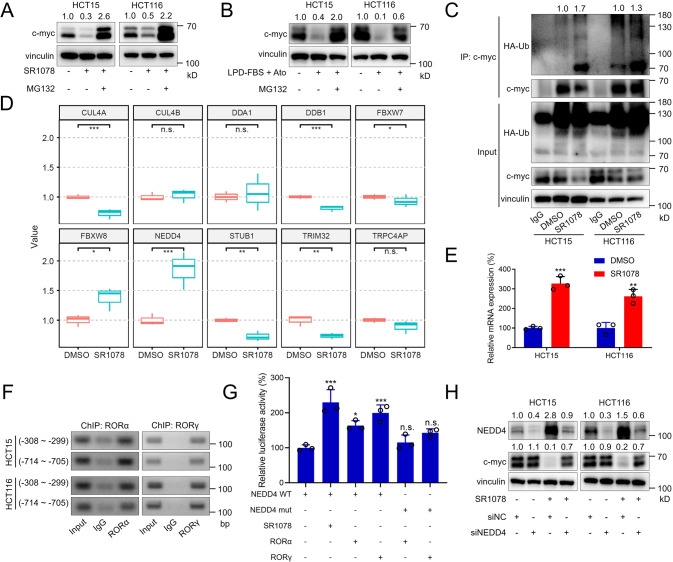
RORα/γ agonists promote c-myc degradation by activating the transcription of the ubiquitinase NEDD4. **A**, **B** Representative immunoblotting of c-myc protein levels in CRC cell lines. In **A** cells were treated with 10 μM SR1078 and/or MG132. In **B** cells were treated with atorvastatin, LPD-FBS and/or MG132 as indicated. **C** Representative immunoblot of ubiquitinated c-myc from the IP assay. CRC cell lines were treated with DMSO or 10 μM SR1078 for 24 h and with MG132 for 12 h. **D** The relative mRNA levels of CUL4A, CUL4B, DDA1, DDB1, FBXW7, FBXW8, NEDD4, STUB1, TRIM32 and TRPC4AP were analyzed in the RNA sequencing data for DMSO-treated and SR1078-treated HCT15 cells. **E** Relative mRNA levels of NEDD4 in CRC cell lines treated with DMSO or 10 μM SR1078 for 24 h (*n* = 3). **F** The binding between RORα/γ and the NEDD4 promoter was assessed with ChIP-PCR. **G** NEDD4 transcriptional activity was assessed by a luciferase assay. Cells were transfected with plasmids containing the wild-type or mutated NEDD4 promoter sequence and with the SR1078 or RORα/γ plasmid as indicated (*n* = 3). **H** Representative immunoblot showing the protein levels of NEDD4 and c-myc in CRC cell lines transfected with NEDD4 siRNA or treated with SR1078 as indicated. The data are presented as the mean ± SD values. ^*^P < 0.05, ^**^P < 0.01, ^***^P < 0.001; Student’s *t*-test in two groups; One-way ANOVA in more than two groups.

C-myc levels in tumour cells are controlled transcriptionally and posttranslationally [33, 34]. As the mRNA levels of c-myc were not changed in our RNA sequencing data (data not shown), we speculated that SR1078 affects c-myc degradation. Accordingly, c-myc is usually degraded by the ubiquitin-proteasome system. Therefore, we added MG132, a proteasome inhibitor, to SR1078-treated cells and observed that the c-myc level was restored (Fig. 5A). Consistent with this result, MG132 also restored the c-myc levels in cells treated with atorvastatin and conditioned medium supplemented with LPD-FBS (Fig. 5B). The immunoprecipitation (IP) assay results proved that SR1078 increased c-myc ubiquitination (Fig. 5C).

To date, the proteins involved in c-myc degradation have been systematically reviewed [35]. We analyzed the expression of the related genes in the RNA sequencing data for DMSO-treated HCT15 cells and SR1078-treated HCT15 cells. Among these genes, NEDD4 was extremely upregulated by SR1078 (Fig. 5D). Then, we confirmed this result in HCT15 and HCT116 cells by quantitative real-time PCR (qRT-PCR) (Fig. 5E). To demonstrate that this effect is mediated transcriptionally at the level of RORE activation, we predicted the binding sites for RORα/γ in the NEDD4 promoter. The results of ChIP assays showed that RORα/γ could bind two of these sites (the negative binding sites are not shown) (Fig. 5F). NEDD4 promoter activity was then analyzed by transfecting cells with a luciferase reporter construct driven by the wild-type NEDD4 promoter fragment (from −2500 to +500 bp) or a mutated fragment (with deletion of the two binding site sequences). SR1078 treatment-induced NEDD4 promoter activity and RORα/γ overexpression increased the activity of the wild-type NEDD4 promoter but not the mutated promoter (Fig. 5G). Thus, the protein levels of NEDD4 were increased by SR1078 treatment, and the decrease in the c-myc level by SR1078 treatment was reversed by NEDD4 silencing (Fig. 5H).

Collectively, these data show that the RORα/γ agonists degrade the c-myc protein by binding to the promoter of the NEDD4 ubiquitinase gene and activating its transcription.

### The combination of atorvastatin and RORα/γ agonists synergistically inhibits CRC cell growth and metastasis in vitro and in vivo

**Fig. 6:**
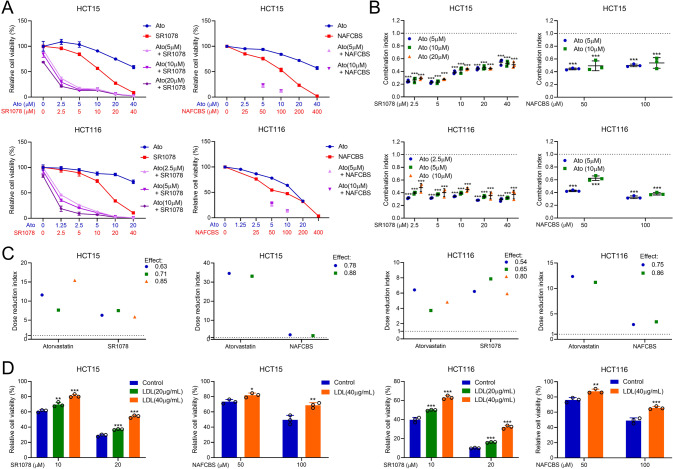
The combination of atorvastatin and SR1078 synergistically inhibits CRC cell viability. **A** The viability of CRC cell lines was evaluated by an ATP assay. Cells were treated with atorvastatin, SR1078 or NAFCBS alone or in combination as indicated for 48 h (*n* = 3). CI (**B**) and DRI (**C**) values for different concentrations of atorvastatin and SR1078/ NAFCBS were calculated using CompuSyn (version 1.1.1) software. **D** Cell viability was measured by an ATP assay. Cells were treated with SR1078 or NAFCBS and different concentrations of LDL (*n* = 3). The viability of cells not treated with a drug was set as 100%. CI Combination index, DRI dose reduction index. The data are presented as the mean ± SD values. ^*^P < 0.05, ^**^P < 0.01, ^***^P < 0.001; Student’s *t*-test in two groups; One-way ANOVA in more than two groups.

**Fig. 7:**
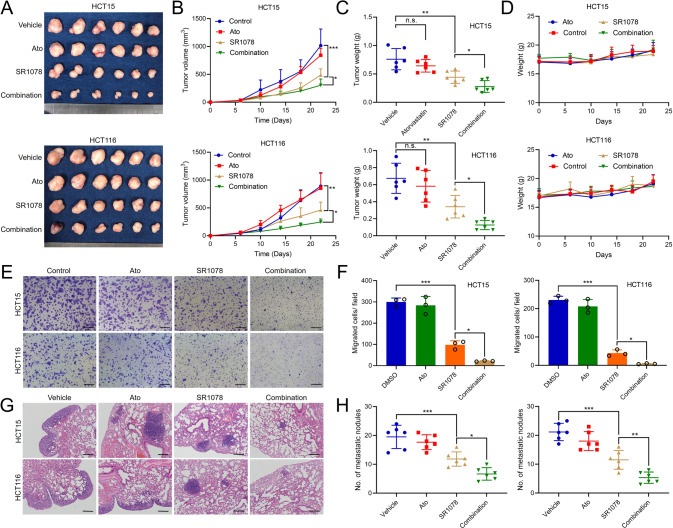
The combination of atorvastatin and SR1078 synergistically inhibits CRC tumour growth and metastasis. **A**–**D** Representative images and growth curves of subcutaneous tumours treated with vehicle, 20 mg/kg atorvastatin, 20 mg/kg SR1078 or the combination in nude mice (*n* = 6). Tumour weights were shown in **C** and mice weights were shown in **D**. **E** The migration ability of CRC cells was evaluated by a transwell assay (*n* = 3). Cells were treated with 5 μM atorvastatin, 5 μM SR1078, or the combination before the assay. The migrated cells were counted after staining with crystal violet. **F** Nude mice were treated with vehicle, 20 mg/kg atorvastatin, 20 mg/kg SR1078 or the combination after tail vein injection of CRC cells (*n* = 6). Lung metastases were evaluated after 8 weeks. Representative H&E staining of lung tissues is shown (left), and the metastatic nodules were counted (right). Scale bars=100 μm. The data are presented as the mean ± SD values. ^*^P < 0.05, ^**^P < 0.01, ^***^P < 0.001; Student’s t-test in two groups; One-way ANOVA in more than two groups.

Since the cholesterol-RORα/γ axis is crucial to CRC progression, we then evaluated whether targeting cholesterol synthesis and RORα/γ can synergistically treat CRC. First, we calculated the Combination index (CI) of atorvastatin and RORα/γ agonists, SR1078 or NAFCBS. We found that these two kinds of drugs had very favourable CI values (approximately 0.2 to 0.6) in different concentration combinations (Fig. 6A, B). Next, we calculated the dose reduction index (DRI) for these two drugs. As shown in Fig. 6C and Supplementary Tables 2, 3, for a given degree of lethal effect, the doses of atorvastatin and SR1078/NAFCBS in combination could be reduced several- to ten-fold compared with the dose required for each drug alone. Conversely, exogenous LDL supplementation decreased the lethal effect of SR1078 or NAFCBS in CRC cells (Fig. 6D). In animal models, for a given dose, atorvastatin alone had only a slight and nonsignificant effect on CRC tumour growth in vivo. However, the combination of atorvastatin and SR1078 effectively inhibited tumour growth more effectively than SR1078 alone (Fig. 7A–C). In addition, the combination did not affect the weight of the mice (Fig. 7D). Moreover, the combination of atorvastatin and SR1078 also synergistically inhibited CRC cell migration in vitro by transwell assays and tumour metastasis in vivo using lung metastasis models (Fig. 7E–H).

To strengthen our results, we explored the synergistic effect of atorvastatin and SR1078 in other cancer types. There were several clinical trials that explored the efficacy of the addition of statins to the standard regents in cancer patients. In small-cell lung cancer, the LUNGSTAR study revealed that pravastatin combined with first-line standard chemotherapy did not benefit patients [36]. In another clinical trial of advanced gastric cancer, Seung Tae Kim et al. showed the addition of simvastatin does not increase progression-free survival [37]. Therefore, we repeated the CI and DRI analysis in a lung cancer cell line A549 and a gastric cancer cell line MGC803. We found atorvastatin and SR1078 also had a synergistic effect in these two cell lines (Supplementary Fig. 5A, B, Supplementary Table 4). Besides, SR1078 increased the mRNA and protein levels of NEDD4, decreased the protein levels of c-myc (Supplementary Fig. 5C, D).

In summary, we found that the cholesterol-RORα/γ axis is crucial for c-myc protein stabilization in CRC cells. Our findings might be extended to other types of tumors. The mechanism is illustrated in the graphical abstract. Cholesterol and its derivatives inhibit RORα/γ-mediated NEDD4 transcription, thus maintaining c-myc levels by reducing its ubiquitination degradation.

## Discussion

We found that RORα/γ are activated in CRC cells treated with atorvastatin and conditioned medium supplemented with LPD-FBS. RORα/γ exerted an inhibitory effect on CRC cell proliferation and invasion by activating NEDD4 transcription, which in turn leads to c-myc degradation. In addition, the combination of RORα/γ agonists and atorvastatin synergistically killed CRC cells and inhibited CRC metastasis, suggesting that targeting the cholesterol-RORα/γ axis is a new therapeutic approach for CRC.

The fast growth of cancer cells requires robust cholesterol metabolism [7]. Oxysterols are produced from cholesterol and then bind to NRs. The relationship between cholesterol metabolites and NRs has been investigated in several kinds of tumours. Among these NRs, LXRs act as sensors of cholesterol, reduce its cellular uptake and enhance its conversion to bile acids [38]. Dysregulated cholesterol metabolism in glioblastoma cells suppresses LXR activity. Conversely, LXR agonists have an antitumour effect on glioblastoma cells by reducing cholesterol [39]. Cai et al. screened 31 small molecules targeting NR family members in TNBC and found that RORγ is a master regulator of cholesterol biosynthesis and is targetable in TNBC [26]. In addition, the cholesterol metabolite 27-hydroxycholesterol can promote prostate cancer cell proliferation by activating ERβ [40]. The dominant NR in CRC is worth exploring. Our evaluation of the levels of these NRs under cholesterol deprivation revealed a crucial role for RORα/γ in the cholesterol regulatory pathway in CRC (Fig. 1A).

Recently, studies on the RORs contributing to cancer progression have been increasing [41]. RORα regulates cellular activities through both canonical and noncanonical pathways [42]. As a TF, RORα binds to ROREs and modulates the transcription of genes involved in cancer cell proliferation and invasion. On the other hand, RORα can directly bind to some proteins, such as β-catenin and p53, and suppress tumour progression [43, 44]. In our study, we identified a new canonical mechanism of RORα-mediated tumour suppression by which the transcription of the ubiquitinase NEDD4 is activated, leading to c-myc degradation. The second ROR family member, RORβ, is a less-discussed molecule in cancer and does not act downstream of cholesterol. In contrast, RORγ is extensively found in various kinds of tumours. In addition to regulating cholesterol biosynthesis in TNBC, RORγ recruits nuclear receptor coactivators 1 and 3 to the RORE in the AR gene and then stimulates AR transcription in CRPC [27]. In our work, we found similar functions of RORα and RORγ in CRC cells. Both can promote NEDD4 transcription and subsequent c-myc protein degradation.

C-myc is an oncogene that is deregulated in most human cancers [45]. Extensive research has shown that oncogenic levels of c-myc reprogram anabolism and catabolism in cancer cells from glycolysis and glutaminolysis to nucleotide and lipid synthesis [46]. For cholesterol metabolism, c-myc binds to the promoters of the key enzymes in cholesterol synthesis and activates SREBP1 transcription, thus inducing fatty acid and cholesterol biosynthesis [47]. Moreover, the metabolic regulators of MYC activity have also been explored. Nutrients such as glucose, glutamine, and their derived metabolites help to maintain high levels of c-myc [48]. Glucose starvation increases c-myc degradation in CRC and hepatocarcinoma cells [49, 50]. Additionally, glutamine deprivation suppresses translation of MYC via changes in intracellular adenosine nucleotide levels [51]. The lipid metabolic regulators of c-myc have been studied less frequently. Intriguingly, we found that cellular cholesterol levels can in turn sustain c-myc levels in CRC cells. The protein levels of c-myc were significantly decreased upon cholesterol deprivation (Fig. 4F). Cholesterol not only is a plasma membrane constituent and a hormone precursor but also plays a key role in controlling c-myc levels and thus regulates diverse aspects of tumour progression.

This study has some potential therapeutic implications. To date, the therapeutic application of statins is limited in various cancer types, including CRC, lung cancer and gastric cancer [12, 36, 37]. Statins have side effects, especially statin-associated muscle symptoms, which limit their clinical use [52, 53]. Additionally, statins only inhibit *de novo* synthesis of cholesterol. Cancer cells can still obtain cholesterol from LDL through the LDL receptor. The LDL levels in serum cannot be reduced too greatly with a safe dose of statins. The combination of statins and RORα/γ agonists targeting the cholesterol-RORα/γ axis offers a solution to this limitation. According to our data, the dose of atorvastatin in combination with RORα/γ agonists required to achieve certain cytotoxicity in cancer cells is several to dozens of folds lower than that required for atorvastatin alone (Fig. 6C, Supplementary Tables 2–4), a very exciting advantage for future application of these drugs in combination. In addition, we found that the cytotoxic mechanism of RORα/γ agonists is mediated through c-myc inhibition not only in CRC cells but also in lung cancer cells and gastric cancer cells. As c-myc is abnormally activated in many types of cancers, its targeting can be a promising approach for cancer treatment. However, the c-myc protein has an “undruggable” structure; thus, its direct targeting is challenging [54]. Many studies have aimed to target c-myc by disrupting MYC transcription or translation, the c-myc/max interaction and c-myc degradation. Our research offers a new strategy for targeting c-myc by inhibiting the cholesterol-RORα/γ axis. This approach may be extended to other c-myc-driven tumour types.

This study has some limitations. First, multiple species of oxysterol exist. We did not distinguish and identify different oxysterols mechanistically involved in the cholesterol-RORα/γ axis in CRC cells. Second, NAFCBS is a novel RORγ agonist through increasing SRC1 recruitment. It is not certain if NAFCBS could activate RORα through the same molecule SRC1. The mechanism of the interaction between these agonists and RORα/γ needs further investigation. Finally, RORα/γ agonists are currently used only for cell and animal experiments, and their clinical use still requires many more pharmacological experiments.

In summary, through a multipronged experimental approach, we identified an important mechanism in the progression of CRC: cholesterol-mediated inhibition of RORα/γ sustains c-myc stability and hence promotes CRC cell growth and metastasis. Targeting the cholesterol-RORα/γ axis with atorvastatin and RORα/γ agonists produces a synergistic effect on CRC cells. These findings indicate the importance of RORα/γ in cholesterol metabolism and reveal RORα/γ agonists as a therapeutic approach for CRC.

## Methods

### Cell lines and human tissue samples

The HCT15, HCT116, HT29, RKO, LS174T, and HCT8 human CRC cell lines, A549 human lung cancer cell line, and MGC803 human gastric cancer cell line were purchased from the American Type Culture Collection (ATCC, Rockville, MD, USA). All the cells were maintained in RPMI 1640 culture medium (HyClone, Logan, UT, USA) supplemented with 10% FBS (Invitrogen, Carlsbad, CA, USA) and 1% penicillin-streptomycin (HyClone). Cells transfected with lentivirus were cultured in a medium supplemented with 2 μg/mL puromycin (T2219, Targetmol, Wellesley Hills, MA, USA). For cholesterol deprivation, cells were cultured in RPMI 1640 culture medium supplemented with lipoprotein-depleted fetal bovine serum (LPD-FBS) (Kalen Biomedical, LLC, USA). All cell lines were regularly tested for mycoplasma contamination. Cells were cultured in an incubator with 5% CO_2_ at 37 °C. Human tissue samples from CRC patients were obtained from Sun Yat-sen University Cancer Center (SYSUCC, Guangzhou, China) between July 2002 and December 2012. The information for two patient-derived xenografts (PDX) in Fig. 2H–J was listed in Supplementary Table 1. The study was approved by the ethics committee of SYSUCC, and informed consent and agreement were obtained from all patients.

### RNA extraction and qRT-PCR

RNA extraction was performed using an RNA purification kit (ESscience Biotech, RN001, Shanghai, China). Then, cDNA was synthesized with PrimeScript™ RT Master Mix (Takara, RR036A, NHK, Japan) from RNA samples (1.0 μg). The samples were mixed with qRT-PCR reagents (GoTaq^®^ qPCR Master Mix, A6002, Promega, Madison, USA) on the PCR plates (409013, NEST Biotechnology, Wuxi, China). Target mRNA levels were measured on Roche light cycler 480 II and normalized to those of β-actin (ACTB). The sequences of the primers are listed in Supplementary Table 5. The primers were synthesized by TsingKe (Beijing, China).

### Western blotting

Cells were harvested, and proteins were extracted using cell lysis buffer (Beyotime Biotechnology, Shanghai, China) containing a protease inhibitor cocktail. The protein concentration was measured with a BCA kit (Thermo Fisher Scientific, Carlsbad, CA, USA). The primary antibodies and their concentrations were as follows: anti-vinculin (Abcam, 1:1000), anti-RORα (Abcam, ab60134, 1:1000), anti-RORγ (Proteintech, 13205-1-AP, 1:1000), anti-c-myc (Abcam, ab32072, 1:1000), anti-HA-Peroxidase (Roche, 12013819001, 1:1000) and anti-NEDD4 (Cell Signaling Technology, CST, #2740, 1:1000). Western blotting was performed as described previously [55]. The gray values of the immunoblots were measured by ImageJ (version 1.48) software (Wayne Rasband, National Institutes of Health, USA). The statistical differences for all the western bot data were shown in Supplementary Figs. 6, 7.

### Gene reporter assay and plasmids transfection

For detection of RORα/γ activity, CRC cells cultured in 96-well plates were transfected with a pGL4.20 construct containing the RORE motif using ViaFect^TM^ Transfection Reagent (Promega, E4892). After transfection, cells were treated with 10 μM atorvastatin (MedChemExpress (MCE), HY-B0589, Monmouth Junction, USA), conditioned medium containing LPD-FBS or an exogenous LDL supplement (40 μg/mL). For detection of NEDD4 promoter activity, we cloned the wild-type NEDD4 promoter region (−2500 bp to +500 bp upstream of the transcription start site) and mutant promoter region (with deletion of the two binding site sequences) into the pGL4.20 plasmid. CRC cells were transfected with the RORα or RORγ plasmid, the wild-type or mutant NEDD4 promoter plasmid and the Renilla luciferase vector using ViaFect^TM^ Transfection Reagent. The luciferase activities were measured with a Dual-Glo^®^ Luciferase Assay System (Promega, E2920) after 36 h of transfection, as described previously [56]. For MYC overexpression, pcDNA3.1-MYC plasmid or the corresponding empty vector were transfected into CRC cells using ViaFect^TM^ Transfection Reagent. All the plasmids and constructs were synthesized by Umine Biotechnology Co., LTD (Guangzhou, China).

### Evaluation of malignant phenotypes in vitro and in vivo

For in vitro assays, CRC cell proliferation was measured with an MTS assay, and CRC cell migration was evaluated with a transwell assay as described previously [55].

For the xenograft experiment, 3-4-week-old female BALB/c athymic nude mice were obtained from Beijing Vital River Laboratory Animal Technology Co., Ltd (Beijing, China) and housed in a pathogen-free animal facility at Sun Yat-sen University. All animal studies were carried out following the guidelines of the Animal Research: Reporting of In Vivo Experiments (ARRIVE) and were approved by the Medical Ethics Committee of Sun Yat-sen University. Randomization was conducted and mice were treated by an unblinded manner. To establish the subcutaneous tumour model, 2 × 10^6^ CRC cells were suspended in 100 μL PBS and then injected subcutaneously (*n* = 5 or 6 per group). Tumour volumes were measured every 3 or 4 days with a caliper and were calculated as follows: volume = length × width^2^ /2. For the drug treatment studies, daily doses of 20 mg/kg atorvastatin, 20 mg/kg SR1078 or the combination were administered via intraperitoneal (IP) injection. After the mice were euthanized, the tumours were excised and weighed. To establish the lung metastasis model, 2 × 10^6^ CRC cells were suspended in 200 μL of PBS and were then injected into nude mice via the tail vein (*n* = 6 per group). The metastatic ability was evaluated after 8 weeks. For the drug treatment studies, mice received 20 mg/kg atorvastatin, 20 mg/kg SR1078 or the combination through i.p. injection every 2 days. The lungs were excised, and the metastatic nodules were counted under a microscope. Metastasis was confirmed by haematoxylin–eosin (H&E) staining. For the PDX assay, the PDX model was established as described previously [57]. SR1078 (20 mg/kg) was administered daily via i.p. injection.

### Combination index (CI) and dose reduction index (DRI) analysis

CRC cells were treated with SR1078, NAFCBS, atorvastatin or the combination at the indicated concentrations for 48 h. Cell survival was assessed with a CellTiter-Glo® Luminescent Cell Viability Assay Kit (Promega, G7570) according to the manufacturer’s instructions. The CI of the drugs was calculated using CompuSyn (version 1.1.1) software (T. C. Chou and N. Martin, Memorial Sloan-Kettering Cancer Center, New York). CI values<1 indicate synergism (the smaller the value, the greater is the degree of synergy), CI values equal to 1 indicate an additive effect, and CI values>1 indicate antagonism. The DRI indicates the fold reduction in the concentration of each drug in combination compared to that of each drug alone needed to achieve a defined effect level. In principle, combinations with a DRI of >1 can be clinically valuable in reducing the risk of developing drug toxicity while retaining therapeutic efficacy.

### Statistics

All data are presented as the mean ± SD of at least three independent experiments. The determination of sample size was depended on the need for statistical power. The immunohistochemical analysis about samples from CRC patients was performed by a blinded manner. Statistical analyses were performed using GraphPad Prism 8.02 (GraphPad Software Inc., San Diego, CA, USA). Gene expression was analyzed using the 2 − ^ΔΔCt^ method. Survival analyses were performed using the Kaplan–Meier method, and differences were evaluated using the log-rank test. Student’s *t*-test was used in two groups, and one-way ANOVA was used in more than two groups. The student’s *t*-test was two-sided. *P* < 0.05 was considered statistically significant. The variance between the groups that are statistically compared is similar.

For further details, see the online supplementary methods section.

## Supplementary information

Supplementary material

Supplementary Table 6
