# Supplementary material for: Targeting the cholesterol-RORα/γ axis inhibits colorectal cancer progression through degrading c-myc
Source: Oncogene. 2022 Oct 31;41(49):5266–78. doi: 10.1038/s41388-022-02515-3 (PMC9718673; doi:10.1038/s41388-022-02515-3)
Supplement: Supplementary file 1 — Supplementary material [file 41388_2022_2515_MOESM1_ESM.pdf]

Supplementary Information Text

Supplementary methods

Lentivirus and adenovirus transfection

Lentiviruses for silencing RORα or RORγ and adenoviruses expressing RORα or RORγ were purchased from OBiO Technology Corp., Ltd (Shanghai, China). The sequences of the shRNAs are listed as follows: RORα, 5'-CCCGACGTCTTCAAATCCT-3', 5'-CACGACGACCTCAGTAACT-3'; RORγ, 5'-GAGCAGATACCCTCACCTA-3', 5'-GTGATCCCTTGCAAAATCT-3'. The two sequences were used as a mixture. CRC cells were infected with lentivirus or adenovirus in the presence of 5 μg/mL polybrene. After puromycin selection for one week, the knockdown and overexpression efficiencies were confirmed by western blot analysis.

SiRNA transfection

SiRNA was used to knock down NEDD4 and MYC gene expression. The siRNA sequences were synthesized by RiboBio Co., Ltd (Guangzhou, China) and are listed as follows: siRNA1 for NEDD4, 5'-GGAAGGACCTATTATGTAA-3'; siRNA2 for NEDD4, 5'-GGAACAACCTACACTTCCT-3'; siRNA3 for NEDD4, 5'-GGAGAATTATGGGTGTCAA-3'; siRNA1 for MYC, 5'-CCTGAGACAGATCAGCAACAA-3'; siRNA2 for MYC, 5'-CAGTTGAAACACAAACTTGAA-3'. CRC cells were transfected with a cocktail containing the three NEDD4 siRNAs or a single MYC siRNA by using Lipofectamine™ RNAiMAX Transfection Reagent (Invitrogen, 13778030) according to the manufacturer's instructions.

RNA sequencing analysis

HCT15 cells were treated with DMSO or 10 μM SR1078 for 24 h in triplicate. Total RNA was extracted using TRIzol reagent following the manufacturer's instructions. A total amount of 3 μg of RNA was used for library preparation. In brief, ribosomal RNA was removed, and sequencing libraries were constructed with an NEBNext® Ultra™ Directional RNA Library Prep Kit for Illumina® (NEB, USA). Then, RNA sequencing was performed by Novogene Co., Ltd (Beijing, China) on the Illumina HiSeq 4000 platform. After removing low quality reads, clean reads were mapped to the hg19 genome with HISAT2 software [1]. The transcription levels (Counts) were quantified with featureCounts tool [2]. Differential expression analysis was performed with DESeq2 to determine the differential abundances of mRNAs between the SR1078 group and DMSO group. Upregulated or downregulated genes were defined as those with $\log_2$(SR1078 group/DMSO group) ≥ 1 or ≤ -1, respectively. The differentially expressed gene list was shown in Supplementary Table 6. Cancer hallmark pathway enrichment analysis was performed via the gene set enrichment analysis (GSEA) method [3]. Transcription factor (TF) enrichment analysis was performed by Enrichr with the consensus TFs from the ChEA and ENCODE databases [4-6]. The RNA-seq data from SR1078 or DMSO-treated HCT15 cells have been deposited in BioProject under accession PRJNA793361.

Immunoprecipitation (IP)

For co-IP analysis of c-myc ubiquitination, CRC cells were transfected with the HA-tagged Ub plasmid (Umine bioTechnology Co., Ltd, Shanghai, China) and were then treated with DMSO or 10 μM SR1078 for 24 h and with MG132 for 12 h. Next, cells were lysed with IP buffer (Beyotime Biotechnology) for 30 min on ice and were centrifuged at 12,000 × g for 10 min at 4°C. The supernatants were incubated with anti-c-myc antibody-conjugated magnetic beads

for 2 h as described in the manufacturer's instructions (MCE). Eluted proteins were analysed by western blotting.

Chromatin immunoprecipitation (ChIP) assay

The ChIP assay was performed with the MAGnify™ Chromatin Immunoprecipitation System (Invitrogen, 492024) following the manufacturer's instructions. In brief, CRC cells were harvested, and the DNA and proteins were then crosslinked with 1% formaldehyde and quenched with glycine. Next, chromatin was sonicated to an average length of 200 to 800 bp. After centrifugation at 12,000 × g, the supernatants were immunoprecipitated with either control IgG or an anti-RORα/γ antibody. Finally, the eluted DNA was amplified with the designed primers specific for the NEDD4 promoter. The sequences of the primers are listed in supplementary table 1. The primers were synthesized by TsingKe.

Extracellular acidification rate (ECAR) and oxygen consumption rate (OCR) measurements

The rates of glycolysis (ECAR) and mitochondrial respiration (OCR) were determined with a Seahorse XF24e extracellular flux analyser (Agilent Technologies, Santa Clara, CA, USA). HCT15 and HCT116 cells were treated with 10 μM SR1078 for 1 d and were then collected for measurement according to the manufacturer's instructions. The ECAR and OCR were analysed with the Seahorse XF Report Generator, and the values were then normalized to the protein levels in each well.

Immunohistochemical analysis (IHC)

Tumour tissues were collected from CRC patients at SYSUCC. Then, the tumour samples were paraffin embedded and sliced into 4-μm sections. IHC analysis was performed using anti-RORα (Abcam, ab60134) or anti-RORγ (Abcam, ab219496) antibodies according to a previously described method [7]. The results were evaluated by two pathologists independently. The final score is obtained by multiplying the staining grades and the proportion of stained cells in all tumour cells. The representative images of different staining grades were shown as Supplementary figure 2.

Supplementary figures

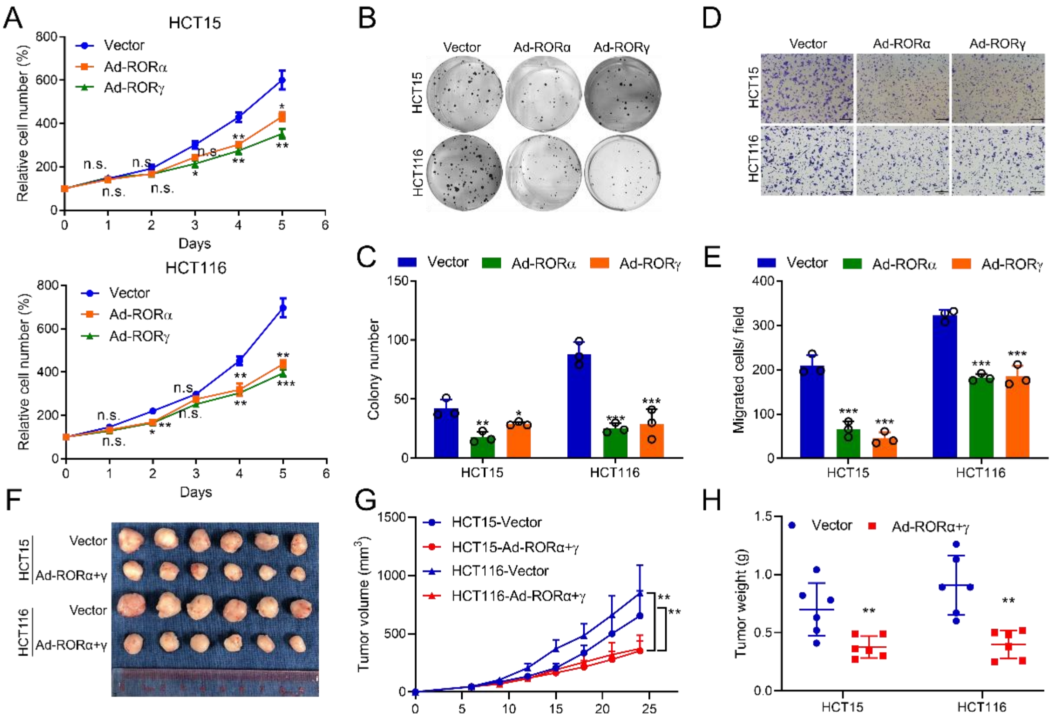

Supplementary figure 1, related to figure 3. RORα/γ inhibit CRC growth and metastasis in vitro and in vivo. (A) Growth curves of Vector, Ad-RORα and Ad-RORγ CRC cells were measured by an MTS assay (n = 3). (B-E) Colony formation assay (B&C) and transwell assay (D&E) results of Vector, Ad-RORα and Ad-RORγ CRC cells (n = 3). Cells were stained with crystal violet after fixation with methanol. The colonies or migrated cells were counted. Scale bars = 200 μm. (F-H) Representative images (F) and growth curves (G) of subcutaneous tumours derived from vector and Ad-RORα+γ cells in nude mice (n = 6). Then, the tumours were weighed (H). The data are presented as the mean ± SD values. **P* < 0.05, ***P* < 0.01, ****P* < 0.001; Student's t-test in two groups; One-way ANOVA in more than two groups.

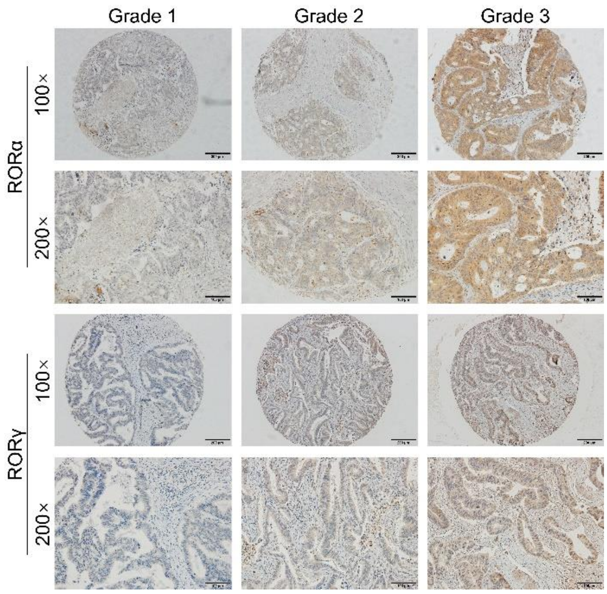

Supplementary figure 2, related to figure 3. The representative images of immunohistochemical analysis. The tumour samples from CRC patients were stained with RORα and RORγ antibody. The representative images with different grades of staining were shown. Scale bars = 200 μm (100×). Scale bars = 100 μm (200×).

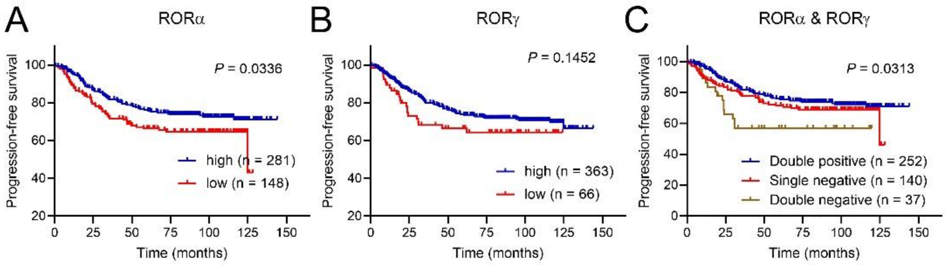

Supplementary figure 3, related to figure 3. Progression-free survival (PFS) analysis in RORα/γ low/high CRC patients. (A&B) Kaplan–Meier estimates of PFS for CRC patients with high or low RORα/γ expression. (C) Kaplan–Meier estimates of PFS for CRC patients with different RORα/γ expressions. Double positive: high levels of RORα and RORγ. Single positive: high levels of RORα and low levels of RORγ, or low levels of RORα and high levels of RORγ. Double negative: low levels of RORα and RORγ. The cut-off points for RORα or RORγ were determined by the Youden index using receiver operating characteristic analysis. log-rank test.

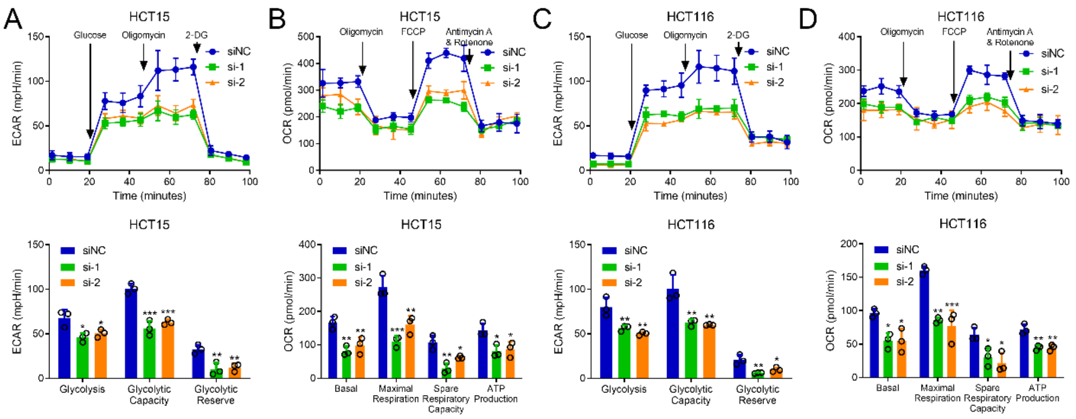

Supplementary figure 4, related to figure 4. Seahorse assay of c-myc-silencing CRC cells. (A-D) HCT15 and HCT116 CRC cells were transfected with siNC or siRNA for MYC. Then, cells were harvested for measurement of the ECAR and OCR using a Seahorse XF24e extracellular flux analyzer (n = 3). The data are presented as the mean ± SD values. *$P < 0.05$, **$P < 0.01$, ***$P < 0.001$; One-way ANOVA.

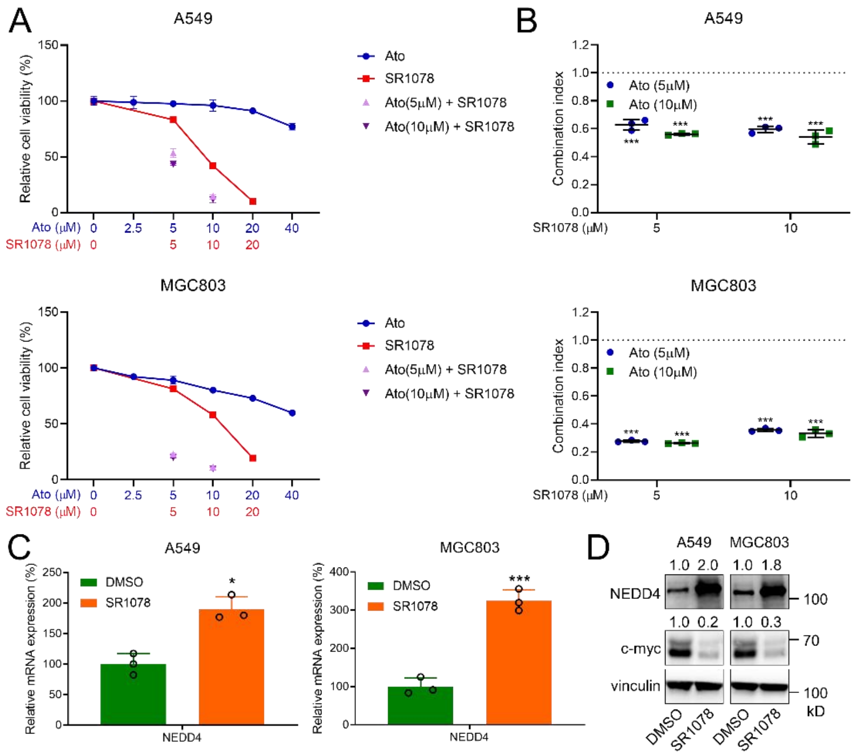

Supplementary figure 5, related to figure 6. SR1078 and/or atorvastatin treatment in A549 lung cancer cell line and MGC803 gastric cancer cell line. (A) The viability of A549 and MGC803 cells was evaluated by an ATP assay. Cells were treated with atorvastatin or SR1078 alone or in combination as indicated for 48 h (n = 3). (B) Combination index (CI) values for different concentrations of atorvastatin and SR1078 were calculated using CompuSyn (version 1.1.1) software. (C) Relative mRNA levels of A549 and MGC803 cells treated with DMSO or 10 μM SR1078 for 24 h (n = 3). (D) Representative immunoblot showing the protein levels of NEDD4 and c-myc in A549 and MGC803 cells treated with SR1078. The data are presented as the mean ± SD values. **P* < 0.05, ****P* < 0.001; Student's t-test in two groups; One-way ANOVA in more than two groups.

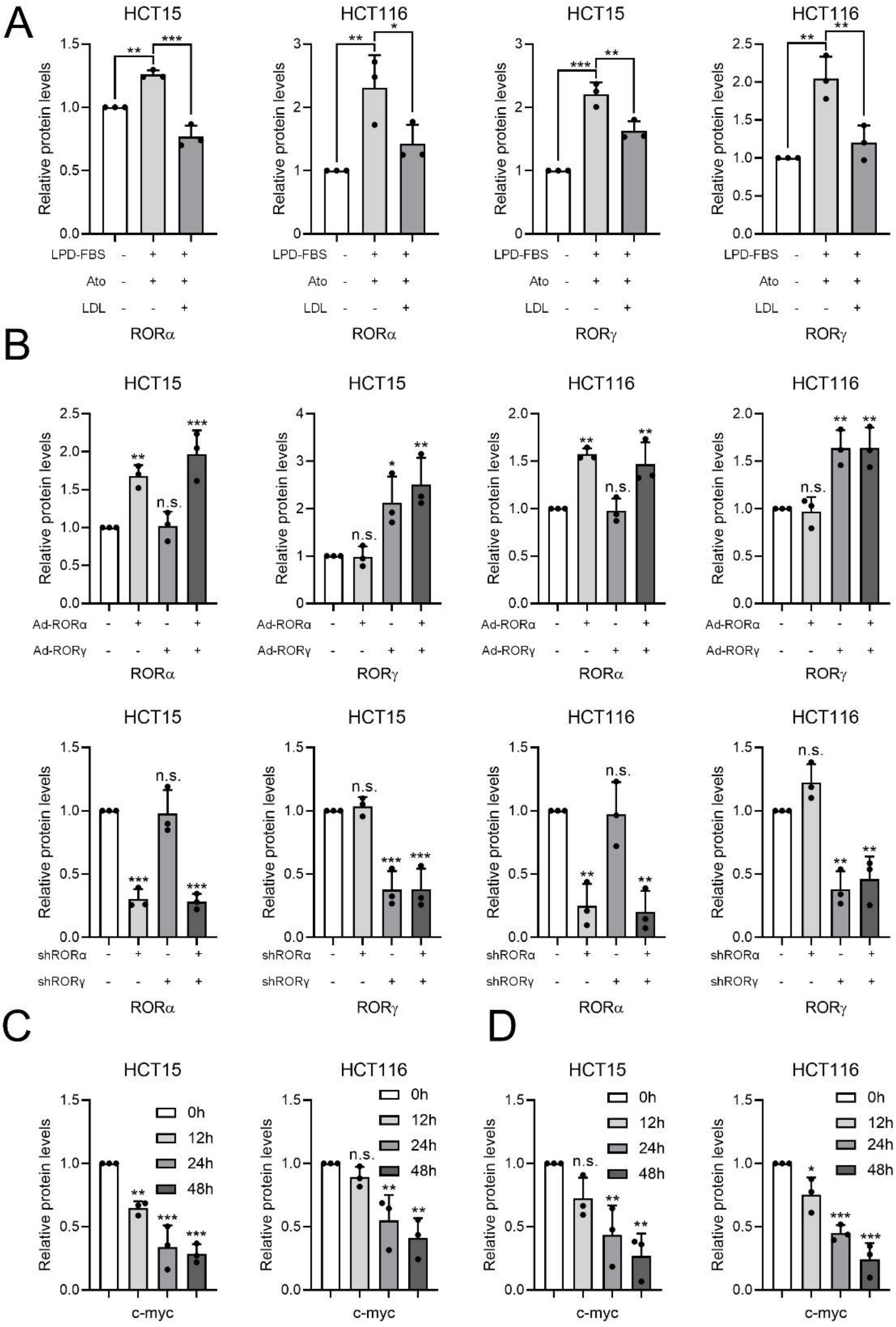

Supplementary figure 6, related to figure 1, 3, and 4. The statistical differences for the western bot data in figure 1, 3 and 4. (A) Related to figure 1C. (B) Related to figure 3A. (C) Related to figure 4D. (D) Related to figure 4E. The data are presented as the mean ± SD values. **P* < 0.05, ***P* < 0.01, ****P* < 0.001; One-way ANOVA.

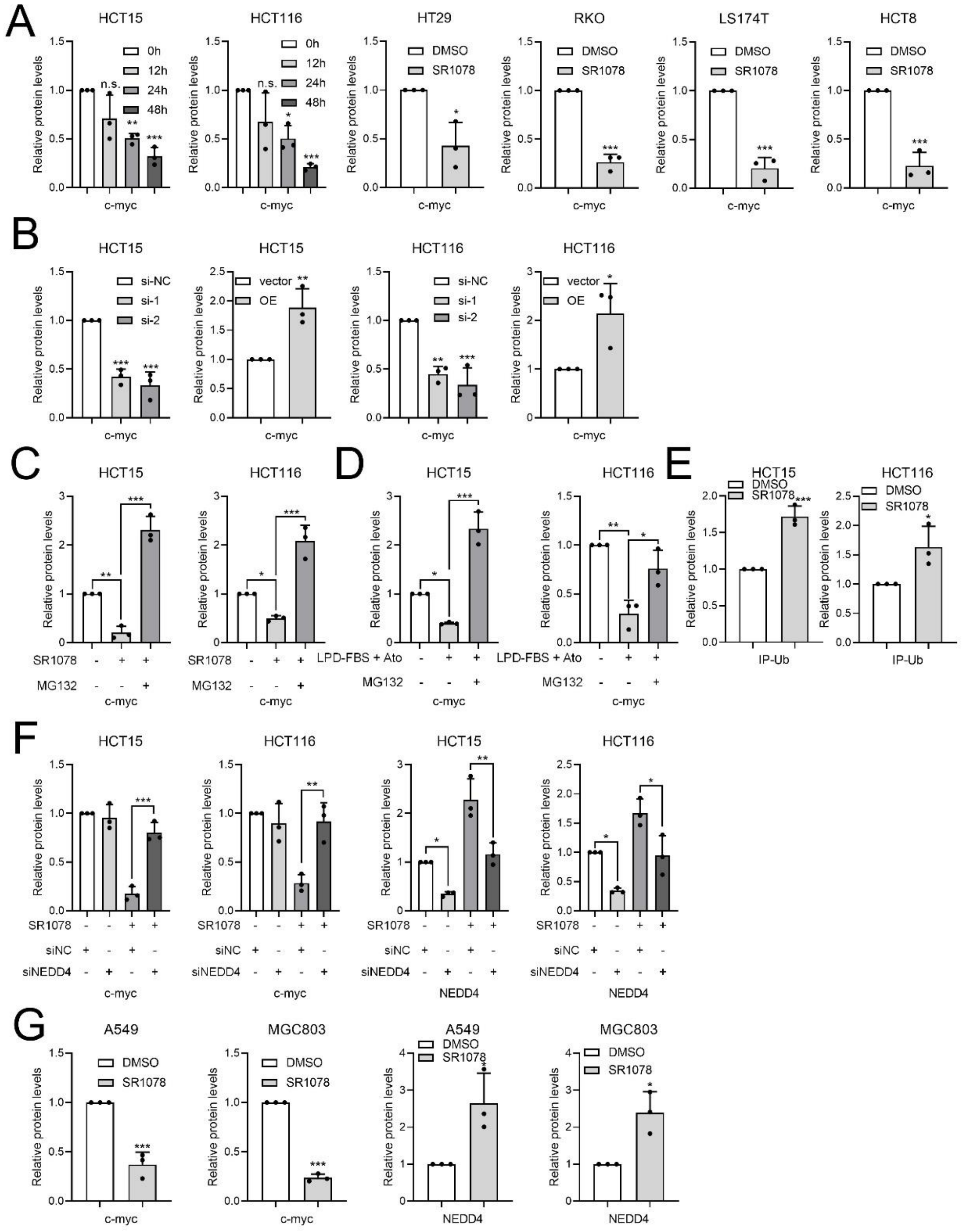

Supplementary figure 7, related to figure 4, 5, and supplementary figure 5. The statistical differences for the western bot data in figure 4, 5, and supplementary figure 5. (A) Related to figure 4F. (B) Related to figure 4K. (C) Related to figure 5A. (D) Related to figure 5B. (E) Related to figure 5C. (F) Related to figure 5H. (G) Related to supplementary figure 5D. The data are presented as the mean ± SD values. **P* < 0.05, ***P* < 0.01, ****P* < 0.001; Student's t-test in two groups; One-way ANOVA in more than two groups.

Supplementary table 1. The patient information for two patient-derived xenografts (PDX).

	Patient#1	Patient#2
Gender	Male	Male
Age	74	77
TNM stage (AJCC)	T2N1M0, stage IIIA	T3N0M0, stage IIA
Tumour site	Rectum	Colon
Differentiation	Moderate	Low
Chemotherapy	Yes	No

Supplementary table 2. Dose reduction index (DRI) for drug combination of Atorvastatin and SR1078 in HCT15 and HCT116 cells.

Cell lines	Drug combination effect	Atorvastatin			SR1078		
		Concentration (μM)		DRI	Concentration (μM)		DRI
		Alone	Mix		Alone	Mix	
HCT15	0.63	57.98	5.00	11.60	15.73	2.50	6.29
	0.71	76.36	10.00	7.64	18.74	2.50	7.50
	0.85	154.36	10.00	15.44	29.34	5.00	5.87
	0.95	367.72	20.00	18.39	50.99	20.00	2.55
HCT116	0.54	16.06	2.50	6.42	15.56	2.50	6.22
	0.65	18.62	5.00	3.72	19.64	2.50	7.86
	0.80	24.16	5.00	4.83	29.63	5.00	5.93
	0.98	55.52	10.00	5.55	110.00	20.00	5.50

DRI represent the fold of dose reduction that is allowed in combination for a given degree of effects as compared with the dose of each drug alone.

Supplementary table 3. Dose reduction index (DRI) for drug combination of Atorvastatin and NAFCBS in HCT15 and HCT116 cells.

Cell lines	Drug combination effect	Atorvastatin			NAFCBS		
		Concentration (μM)		DRI	Concentration (μM)		DRI
		Alone	Mix		Alone	Mix	
HCT15	0.78	172.95	5.00	34.59	120.13	50.00	2.40
	0.88	331.06	10.00	33.11	196.16	100.00	1.96
HCT116	0.75	61.80	5.00	12.36	145.04	50.00	2.90
	0.86	112.06	10.00	11.21	344.41	100.00	3.44

Supplementary table 4. Dose reduction index (DRI) for drug combination of Atorvastatin and SR1078 in A549 and MGC803 cells.

Cell lines	Drug combination effect	Atorvastatin			SR1078		
		Concentration (μM)		DRI	Concentration (μM)		DRI
		Alone	Mix		Alone	Mix	
**A549**	0.47	123.78	5.00	24.76	8.52	5.00	1.70
	0.85	662.37	5.00	132.48	17.01	10.00	1.70
	0.57	177.71	10.00	17.77	9.89	5.00	1.98
	0.88	848.28	10.00	84.83	18.84	10.00	1.88
**MGC803**	0.77	347.45	5.00	69.49	19.07	5.00	3.81
	0.89	1033.45	5.00	206.69	28.37	10.00	2.84
	0.80	442.32	10.00	44.23	20.83	5.00	4.17
	0.90	1292.87	10.00	129.29	30.78	10.00	3.08

Supplementary table 5. Primers used for qRT-PCR and ChIP-PCR. Related to the methods and supplementary methods.

RORA	F: GTCAGCAGCTTCTACCTGGAC<br>R: GTGTTGTTCTGAGAGTCAAAGGCACG
RORC	F: AGAAGACCCACACCTCACAAA<br>R: CCTCACAGGTGATAACCCCG
ESR1	F: GAAAGGTGGGATACGAAAAGACC<br>R: GCTGTTCTTCTTAGAGCGTTTGA
NR1H1	F: GGGCTTCTAGGGACCTCAGT<br>R: TGAGGCCTCTGTCCTGAACT
NR1H3	F: ACACCTACATGCGTCGCAAG<br>R: GACGAGCTTCTCGATCATGCC
ACTB	F: CATGTACGTTGCTATCCAGGC<br>R: CTCCTTAATGTCACGCACGAT
NEDD4	F: TCAGGACAACCTAACAGATGCT<br>R: TTCTGCAAGATGAGTTGGAACAT
NEDD4-promoter-1	F: GGAGGTGGGTGGTTGACG<br>R: GGAGATTGGATTGTTACTGGTG
NEDD4-promoter-2	F: GATCGCAGCAGTCTTGGAC<br>R: ATAAATTGTGGCCTGGGTG

SI References

1. Kim, D., B. Langmead, and S.L. Salzberg, *HISAT: a fast spliced aligner with low memory requirements.* Nat Methods, 2015. **12**(4): p. 357-60.
2. Liao, Y., G.K. Smyth, and W. Shi, *featureCounts: an efficient general purpose program for assigning sequence reads to genomic features.* Bioinformatics, 2014. **30**(7): p. 923-30.
3. Subramanian, A., et al., *Gene set enrichment analysis: a knowledge-based approach for interpreting genome-wide expression profiles.* Proc Natl Acad Sci U S A, 2005. **102**(43): p. 15545-50.
4. Kuleshov, M.V., et al., *Enrichr: a comprehensive gene set enrichment analysis web server 2016 update.* Nucleic Acids Res, 2016. **44**(W1): p. W90-7.
5. Davis, C.A., et al., *The Encyclopedia of DNA elements (ENCODE): data portal update.* Nucleic Acids Res, 2018. **46**(D1): p. D794-D801.
6. Lachmann, A., et al., *ChEA: transcription factor regulation inferred from integrating genome-wide ChIP-X experiments.* Bioinformatics, 2010. **26**(19): p. 2438-44.
7. Wang, Y.N., et al., *CPT1A-mediated fatty acid oxidation promotes colorectal cancer cell metastasis by inhibiting anoikis.* Oncogene, 2018. **37**(46): p. 6025-6040.
